# Retraction: LRP6 knockdown ameliorates insulin resistance via modulation of autophagy by regulating GSK3β signaling in human L02 hepatocytes

**DOI:** 10.3389/fendo.2025.1751575

**Published:** 2025-11-27

**Authors:** Frontiers Editorial Office

The journal retracts the February 19 2019 article cited above.

Following publication, concerns were raised regarding the integrity of the images in the published figures. The authors failed to provide a satisfactory explanation during the investigation, which was conducted in accordance with Frontiers’ policies.

This retraction was approved by the Chief Executive Editor of Frontiers. The authors received a communication regarding the retraction and had a chance to respond. This communication has been recorded by the publisher.

Frontiers would like to thank the users on PubPeer for bringing the published article to our attention.

